# Compliance of systematic reviews in veterinary journals with Preferred Reporting Items for Systematic Reviews and Meta-Analysis (PRISMA) literature search reporting guidelines

**DOI:** 10.5195/jmla.2017.246

**Published:** 2017-07-01

**Authors:** Lorraine C. Toews

## Abstract

**Objective:**

Complete, accurate reporting of systematic reviews facilitates assessment of how well reviews have been conducted. The primary objective of this study was to examine compliance of systematic reviews in veterinary journals with Preferred Reporting Items for Systematic Reviews and Meta-Analysis (PRISMA) guidelines for literature search reporting and to examine the completeness, bias, and reproducibility of the searches in these reviews from what was reported. The second objective was to examine reporting of the credentials and contributions of those involved in the search process.

**Methods:**

A sample of systematic reviews or meta-analyses published in veterinary journals between 2011 and 2015 was obtained by searching PubMed. Reporting in the full text of each review was checked against certain PRISMA checklist items.

**Results:**

Over one-third of reviews (37%) did not search the CAB Abstracts database, and 9% of reviews searched only 1 database. Over two-thirds of reviews (65%) did not report any search for grey literature or stated that they excluded grey literature. The majority of reviews (95%) did not report a reproducible search strategy.

**Conclusions:**

Most reviews had significant deficiencies in reporting the search process that raise questions about how these searches were conducted and ultimately cast serious doubts on the validity and reliability of reviews based on a potentially biased and incomplete body of literature. These deficiencies also highlight the need for veterinary journal editors and publishers to be more rigorous in requiring adherence to PRISMA guidelines and to encourage veterinary researchers to include librarians or information specialists on systematic review teams to improve the quality and reporting of searches.

## INTRODUCTION

Systematic reviews are a type of research synthesis that uses a clearly formulated question and systematic, explicit, and reproducible methods to identify, select, and critically appraise all relevant published and unpublished studies and to collect and analyze data from the studies included in the review. Since an individual biomedical study cannot provide definitive evidence, well-conducted systematic reviews are a powerful and reliable form of evidence because they contextualize and integrate individual studies within the full body of available research on a topic [1].

Because the literature search is the data collection method in a systematic review and the search results form the evidence base of the review, it is critical that the search is reported in sufficient detail so that it can be replicated. Unfortunately, incomplete and inaccurate reporting of systematic review literature searches [2, 3] is part of a larger, widespread problem of poor reporting of all types of biomedical research [4–6]. Poor reporting of biomedical research has serious consequences, making it difficult or impossible to assess the quality of, replicate the study, or use the study in clinical decision making or in subsequent systematic reviews [7]. To address this problem and improve the reproducibility of published research, biomedical journal editors and research funding bodies have supported the development of research reporting guidelines that specify a baseline of information required for a complete and transparent account of the conduct and findings of research studies [8]. The reporting guideline for systematic reviews and meta-analyses is Preferred Reporting Items for Systematic Reviews and Meta-Analysis (PRISMA), published in 2009 [4].

While reporting a systematic review and actually conducting the review are distinct processes, they are closely interrelated. Complete, accurate reporting helps determine how well a systematic review was conducted, whereas incomplete, inaccurate reporting raises questions about the conduct, quality, and reliability of a systematic review [4, 7, 9, 10]. A recent study found that librarian participation in internal medicine systematic reviews as coauthors who developed, conducted, and reported the search methodology was associated with higher quality reporting of searches and better search reproducibility [11]. By contrast, the quality of reporting of literature searches has not been evaluated for veterinary medicine systematic reviews, and the participation of librarians as coauthors of veterinary systematic reviews appears to be much less common.

The first objective of this study was to address this gap in the literature by examining the compliance of literature searches in veterinary systematic reviews with PRISMA reporting guidelines and examining what could be determined about the completeness, bias, and reproducibility of the reported searches. The second objective of this study was to examine reporting of the credentials and contributions of those involved in the reviews. The findings of this study have implications for the role that librarians can play in supporting the production of veterinary systematic reviews. Whereas a previous study found that librarians have played an important role in improving the quality of systematic reviews in the human medicine literature [11], librarians have a significant opportunity to play a comparable role in veterinary systematic reviews by using their expertise to ensure the quality, reproducibility, and strong reporting of search methodologies.

## METHODS

A sample of systematic reviews in veterinary journals was obtained by searching PubMed for titles from Ugaz’s “Basic List of Veterinary Medical Serials, Third Edition” [12], and limiting search results to studies published between 2011 and 2015. Since PubMed does not have a publication type that indexes all types of systematic reviews, search results were limited to those tagged with the PubMed publication type meta-analysis or to studies with the phrase “systematic review” in the title or abstract of the PubMed record. The abstracts of these records were reviewed, and only records whose authors explicitly identified their studies as systematic reviews were included in this study. Authors’ claims that a study was a systematic review were accepted at face value, and no further analysis of these records was done. From these results, reviews from journals in 12 veterinary medicine specialty areas were selected using the subject categories listed in the appendix of the Ugaz study [12], resulting in a final sample of 75 systematic reviews (detailed in the supplemental appendix). The sample size was not predetermined; rather, the goal was to include a variety of veterinary specialty areas. Reporting in the full-text of each review in the final sample was checked against PRISMA checklist search methods items 7 and 8 [4].

## RESULTS

### Information sources: databases

All of the reviews reported a list of the databases that reviewers searched (detailed in the supplemental appendix). As shown in Table 1, the average number of databases searched per review was 4, but 9% of reviews searched only 1 database. Although PubMed and CAB Abstracts were the most common databases searched, over one-third (37%) of reviews did not search CAB Abstracts. One-quarter (24%) of reviews used the Google Scholar search engine, and 5 of these reviews searched only 1 other database in addition to Google Scholar. Twelve (16%) reviews reported that they searched both PubMed and MEDLINE, but only 5 of these reviews noted which vendor search platform they used in searching MEDLINE.

**Table 1:** Literature search reporting characteristics, systematic reviews sample (n=75)

	n	(%)
Databases searched		
PubMed	60	(80%)
CAB Abstracts	47	(63%)
MEDLINE	20	(27%)
Web of Science	19	(25%)
Google Scholar	18	(24%)
Agricola	17	(23%)
Scopus	16	(21%)
Number of databases searched		
1 database	7	(9%)
2 databases	16	(21%)
3 databases	19	(25%)
4 databases	11	(15%)
5 or more databases	22	(29%)
Average number of databases searched=4		
Grey literature		
No grey literature search reported	45	(60%)
Unspecified grey literature searched	9	(12%)
Conference proceedings, named	7	(9%)
Conference proceedings, not named	5	(7%)
Theses, dissertation	5	(7%)
Government agency reports	4	(5%)
Excluded grey literature	4	(5%)
Search strategy		
No search strategy reported	8	(11%)
Search terms listed	63	(84%)
Full electronic database line-by-line strategy	4	(5%)
Credentials of searcher		
Not reported	51	(68%)
Affiliated with a library, but details not specified	4	(5%)
Author, not identified as librarian or information specialist	14	(19%)
Librarian or information specialist	6	(8%)
Contributions to search process		
Not reported	45	(60%)
Assisted, but role not specified	13	(17%)

### Information sources: grey literature

Conference papers and unpublished clinical trials are important types of grey literature in veterinary systematic reviews. Only 16% of reviews in this study reported searching for conference proceedings. Over half of the reviews (60%) did not report any search for grey literature, and a further 5% of reviews reported that they chose to exclude grey literature.

### Search strategies

Because 11% of reviews in this study did not report any search strategy and 84% of reviews reported search terms but not sufficient other details necessary to replicate the search, fully 95% of reviews did not report a reproducible search strategy.

### Credentials of searchers and contributions to the search process

The majority of reviews did not report either the credentials of the persons who planned and conducted the literature search for the review (73%) or the specific contributions made by persons who were involved in the search process (77%).

## DISCUSSION

PRISMA checklist item 7 states that systematic reviews should “describe all information sources (such as databases…) in the search” [4]. Systematic reviews require a comprehensive search for published and unpublished studies in order to minimize bias; therefore, for most review topics, it is necessary to search multiple research databases to ensure that all of the relevant literature is retrieved [2, 13–15]. The appropriate databases to search will vary with the topic of the review, but failure to search multiple databases can increase the risk that relevant studies will be missed, which could bias the outcome of the review [16, 17]. As 9% of the reviews in this study sample searched only 1 database, readers of these studies cannot have confidence that these reviews retrieved all the relevant literature. Since CAB Abstracts indexes the veterinary medicine journal literature more comprehensively than any other research database [18], the 37% of reviews that did not search CAB Abstracts might have missed relevant research that could affect the outcome of the review. One-quarter of the reviews searched Google Scholar, and 5 of these reviews searched only 1 other database in addition to Google Scholar. Several studies have identified problems with using Google Scholar for systematic review searching. Unlike curated databases such as PubMed and CAB Abstracts, Google Scholar does not publish lists of its journal and grey literature source content, so it is impossible to determine what proportion of the biomedical literature it covers. Also, Google Scholar does not publish its search algorithms [19], its search algorithms change without notice, and it displays only the first 1,000 hits of a search, so reproducibility of search strategies with consistent results is not possible [20]. As a result, the completeness and bias of Google Scholar searches cannot be evaluated in the way that it can be for traditional curated databases such PubMed and CAB Abstracts.

The main reason for systematic reviews to conduct a search of the grey literature is to counteract publication bias [21]. Publication bias is a longstanding pattern in the biomedical literature where studies with positive, statistically significant results are more likely to be published than studies with negative or statistically insignificant results. Because of this bias, systematic reviews based only on published studies can overestimate the effectiveness of an intervention [22]. In the medical literature, McAuley et al. found that excluding grey literature from systematic reviews resulted in an overestimate of the effect of the intervention by an average of 12% [23], and Hopewell et al. found a 9% exaggeration of intervention effects [24]. It is not clear whether comparable patterns of overestimation of intervention effects are present in veterinary medicine systematic reviews, but this possibility exists, particularly since a much lower percentage of veterinary conference abstracts are later published in the journal literature than is the case in human medicine [25]. A study of swine and bovine vaccine trial conference papers found that only 5.6% of swine trial conference abstracts and 9.2% of bovine trial conference abstracts were ultimately published in the journal literature [26]. By contrast, a systematic review of conference abstract publication rates in human medicine found that 63% of abstracts of clinical trials presented at conferences were later published as journal articles, and subsequent publication was associated with positive results [25]. It is not clear whether subsequent publication of veterinary trial conference abstracts is associated with positive results [26]. What is clear, however, is that a large body of unpublished veterinary studies with the potential to affect the outcome of the review were excluded from 65% of the systematic reviews in this study, raising serious concerns about bias and potential overestimation of intervention effect in these reviews.

PRISMA checklist item 8 states that systematic reviews should “present [the] full electronic search strategy for at least one database, including any limits used, such that it could be repeated” [4]. Reproducibility is an essential characteristic of all reliable primary biomedical research as well as research synthesis reports such as systematic reviews. Because the studies retrieved by the literature search form the evidence base for a systematic review, it is essential that the search strategy, which is the data collection method for reviews, be reported in sufficient detail to enable the search to be replicated and evaluated [2, 27]. A list of search terms is not sufficient to enable accurate replication of a literature search. Rather, only the exact line-by-line electronic database search strategy contains the level of detail required to replicate the search to evaluate its completeness and detect search errors. The full line-by-line electronic database search history reveals strategies that have a significant impact on both the completeness of the search and the relevance of those records to the review topic, including whether both text words and subject headings were used [2, 13, 16], whether subject headings were exploded [2, 13], and which database record fields were searched. It also reveals search errors such as incorrect use of Boolean and proximity operators [2], incorrect search set combinations, and spelling and truncation errors [28]. Because the majority of reviews (95%) did not report a reproducible search strategy, readers of these reviews would not be able to evaluate the searches for completeness, bias, or search errors.

Although reporting the credentials of the searchers and their contributions to the search is not required by the PRISMA guidelines, the International Committee of Medical Journal Editors strongly encourages biomedical journal editors to require reporting of the contributions made by each person involved in conducting a study [29]. The majority of reviews did not report either the credentials of the persons who planned and conducted the literature search for the review (73%) or the specific contributions made by persons involved in the search process (77%). Because the literature search is the data acquisition method in a systematic review, it would be beneficial for readers of a review to know both the credentials and specific contributions of the persons who were involved in developing and implementing the search methodology.

The *Cochrane Handbook,* which is widely regarded as the gold standard for systematic review search methodology, recommends that systematic review authors seek guidance from a health care librarian or information specialist in planning and conducting the search [13]. Health sciences librarians have training as expert searchers and skills in forming review questions, selecting search terms, selecting databases, crafting high recall searches, managing references, and reporting search methodology to the systematic review process [30–32]. Studies show that systematic reviews in which a librarian or information specialist was a coauthor had literature searches that were more comprehensive [15], had fewer substantive errors [33], were better reported [34], and were more likely to be reproducible [15, 34]. Studies also show that having a second, independent librarian or information specialist peer review the search strategy using a validated tool such as the Peer Review of Electronic Search Strategies (PRESS) guideline “can improve the quality and comprehensiveness of the search and reduce errors” [35] and ultimately improve the overall quality of the review [36].

This study reviewed a relatively small number of systematic reviews from twenty-four veterinary journals, and the reviews in the sample were not randomly selected, so results might not be generalizable. To obtain more definitive results, further research using a larger, randomly selected sample is needed. A majority of reviews in this study had significant deficiencies in reporting the literature search in accordance with PRISMA guidelines. These deficiencies raise questions about how these review literature searches were conducted and ultimately raise serious doubts about the validity and reliability of reviews that are based on a potentially biased and incomplete body of literature. These deficiencies in reporting also highlight the need for veterinary journal editors and publishers to be more rigorous in requiring adherence to the PRISMA guideline and to encourage veterinary researchers to include librarians or information specialists on systematic review teams to improve the quality of review searches as well as the reporting of those searches [26, 34, 37].

## SUPPLEMENTAL FILE

Appendix Final sample of seventy-five systematic reviews
